# Development and validation of a predictive nomogram for differentiating diabetic nephropathy from non-diabetic nephropathy in patients with T2DM: a multicenter study

**DOI:** 10.3389/fnut.2025.1605841

**Published:** 2025-06-02

**Authors:** Zishan Lin, Tao Hong, Wenfeng Wang, Shidong Xie, Caiming Chen, Feng Yang, Dewen Jiang, Jianxin Wan, Zugang Xie, Yanfang Xu

**Affiliations:** ^1^Department of Nephrology, Blood Purification Research Center, the First Affiliated Hospital, Fujian Medical University, Fuzhou, China; ^2^Research Center for Metabolic Chronic Kidney Disease, the First Affiliated Hospital, Fujian Medical University, Fuzhou, China; ^3^Department of Nephrology, National Regional Medical Center, Binhai Campus of the First Affiliated Hospital, Fujian Medical University, Fuzhou, China; ^4^Department of Nephrology, Fuzhou No. 1 Hospital Affiliated with Fujian Medical University, Fuzhou, China; ^5^Department of Nephrology, Longyan People Hospital of Fujian, Longyan, China

**Keywords:** type 2 diabetes mellitus, diabetic nephropathy, diagnostic model, nomogram, triglyceride

## Abstract

**Background:**

Type 2 diabetes mellitus (T2DM) significantly exacerbates the global health burden, with diabetic nephropathy (DN) emerging as one of the most common causes of chronic kidney disease. In T2DM patients with kidney disease, it is particularly important to distinguish DN from non-diabetic nephropathy (NDN), as treatment strategies differ markedly. However, the gold standard, renal biopsy, is often impractical due to its invasive nature. This multicenter study aims to develop a non-invasive diagnostic model to distinguish DN from NDN in T2DM patients.

**Methods:**

From January 2014 to December 2023, T2DM patients undergoing percutaneous renal biopsies at three hospitals in Fujian were enrolled. The model was formulated using logistic regression analysis based on clinical and laboratory parameters. A visual predictive nomogram was developed and subsequently evaluated for its predictive performance.

**Results:**

A total of 292 patients were included, with 164 diagnosed with DN and 128 with NDN. Diabetic retinopathy, duration of diabetes, HbA1c, systolic blood pressure, neutrophil-to-lymphocyte ratio, kidney volume, triglycerides, estimated glomerular filtration rate, and urinary red blood cell count were identified as independent predictors of DN. A nomogram was then constructed. The model demonstrated high diagnostic accuracy with an AUC of 0.941, validated by an independent cohort yielding an AUC of 0.923. Calibration curves showed good agreement between predicted and actual outcomes, and decision curve analysis confirmed notable clinical utility.

**Conclusion:**

The developed model offers a non-invasive, reliable alternative to renal biopsy for distinguishing between DN and NDN in T2DM patients. This tool proves especially valuable in clinical settings where renal biopsy is impractical, helping guide more appropriate treatment decisions.

## 1 Introduction

Type 2 diabetes mellitus (T2DM) and its complications significantly exacerbate the global burden of mortality and disability (1). With changes in dietary structures and lifestyle habits, the prevalence of T2DM globally is dramatically increasing (1, 2). Diabetic nephropathy (DN), as one of the most common complications of T2DM, has seen an increase in incidence with the rising prevalence of diabetes and improvements in diagnostic screening (3, 4). For instance, in China, glomerulonephritis historically held the distinction as the most prevalent form of kidney disease. Yet, over the recent decade, DN has emerged to claim the forefront, becoming the leading cause of renal pathology (5).

It is imperative to recognize that the spectrum of kidney disease in patients with T2DM extends beyond DN. Indeed, diabetic individuals may manifest a variety of nephropathies, including but not limited to, membranous nephropathy (MN) and IgA nephropathy. Significantly, non-diabetic nephropathy (NDN) accounts for a substantial portion of renal pathologies in this demographic. Existing research indicates that such conditions comprise at least 50% of the renal disorders observed in patients with T2DM who were suspected of having NDN and underwent renal biopsy (6, 7). Presently, the cornerstone of DN management involves rigorous regulation of glycemia and hypertension, though outcomes frequently fall short of expectations (3, 8). In contrast, therapeutic strategies for NDN necessitate a tailored approach, contingent upon the specific renal pathology, thereby diverging significantly from DN treatment paradigms. Thus, the precise identification of NDN among T2DM patients presenting with renal disease is crucial.

Presently, renal biopsy stands as the definitive criterion for distinguishing DN from NDN. Yet, the invasive nature of this procedure, alongside contraindications in patients with coagulopathy, severe renal dysfunction, or psychiatric conditions, limits its applicability (9). Moreover, the capability to perform renal biopsies varies across healthcare facilities, with many primary care institutions lacking this technical expertise. Additionally, in low-resource settings, renal biopsies may be restricted due to financial constraints, lack of access to specialized healthcare, and logistical challenges.

Therefore, there is an urgent need to develop non-invasive alternative diagnostic methods that provide healthcare providers with an accessible and affordable way to differentiate DN from NDN. There have been studies that worked on creating predictive models for DN based on clinical variables. However, most of these studies were single-center with smaller sample sizes, fewer included variables, lower model performance, and often employed non-visualized models (10–15). This study is a multicenter study aimed at developing a non-invasive, user-friendly diagnostic model for differentiating between DN and NDN in patients with T2DM who have renal impairment.

## 2 Methods

### 2.1 Patient

**Figure 1:**
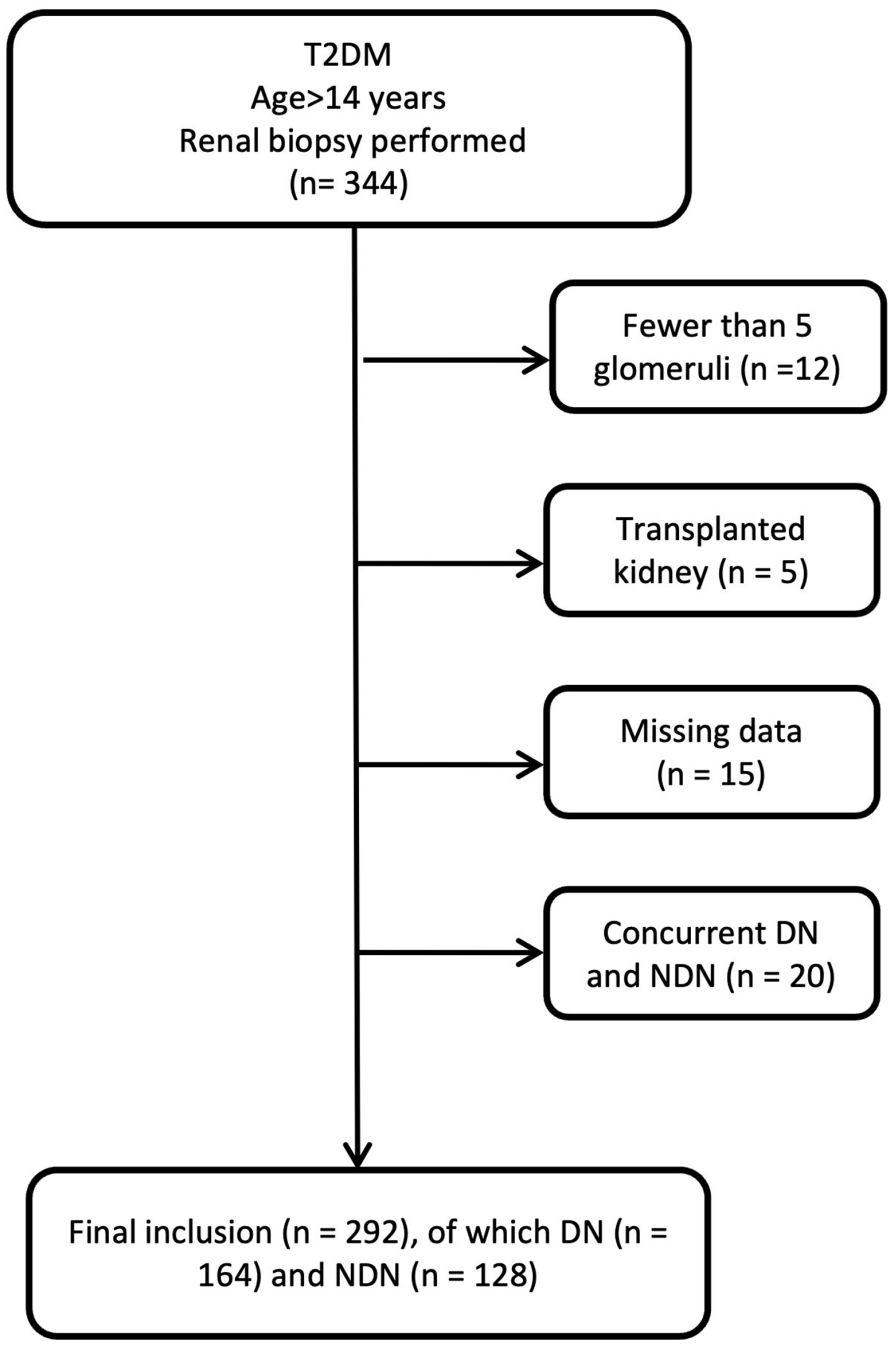
Flowchart of patient selection.

To develop the diagnostic model, we enrolled patients diagnosed with T2DM who underwent percutaneous renal biopsy between January 1, 2014 and May 31, 2023, at the First Affiliated Hospital of Fujian Medical University, Fuzhou NO.1 Hospital Affiliated with Fujian Medical University, and Longyan People Hospital of Fujian. For model validation, we established a validation cohort consisting of T2DM patients who received percutaneous renal biopsies between June 1, 2023, and December 31, 2023, at the aforementioned institutions. The inclusion criteria were that patients be aged over 14 years, diagnosed with T2DM, and have undergone renal biopsy. As previously reported, the indications for renal biopsy in our study were T2DM patients presenting with proteinuria that could not be explained by diabetes (e.g., sudden increase), significant proteinuria with normal renal function, significant hematuria, sudden deterioration of renal function, and absence of diabetic retinopathy (16). Actually, these patients exhibited varying degrees of NDN indicators, prompting the renal biopsy. Exclusion criteria included biopsy samples revealing fewer than five glomeruli within the renal tissue or diagnoses of concurrent DN and NDN (Figure 1). In the biopsy samples of 12 patients, there were fewer than five glomeruli. Among them, 7 patients were diagnosed with DN, 5 patients with NDN, and 2 patients with membranous nephropathy. None of these patients were diagnosed as healthy. All participants provided informed consent prior to their renal biopsy.

### 2.2 Data collection and definitions

This study entailed the comprehensive collection and analysis of patient data at the time of renal biopsy, including demographic information, clinical presentations, medical histories (duration of kidney disease was defined based on both clinical assessment and laboratory confirmation), laboratory and radiological findings, and renal biopsy pathology reports. The estimated glomerular filtration rate (eGFR) was calculated employing the CKD-EPI formula. The renal volume was calculated in cubic centimeters using the ellipsoid formula: volume = length × width × depth × 0.523. Renal biopsy examinations included light microscopy, immunofluorescence, and electron microscopy. Each specimen underwent evaluation by two seasoned renal pathologists. In instances of diagnostic divergence, consultation with a third pathologist was pursued to guarantee diagnostic accuracy. The diagnosis of DN was based on characteristic pathological findings, such as mesangial expansion, nodular glomerulosclerosis (Kimmelstiel-Wilson nodules), and glomerular basement membrane thickening. Hyalinosis of afferent and efferent arterioles, which is typical of DN, was also considered (17).

Nephrotic syndrome was characterized by a urinary protein excretion rate exceeding 3.5 g per day and serum albumin levels falling below 30 g/L. The diagnosis of diabetic retinopathy hinged on the outcomes of fundus examinations conducted by ophthalmologists, aligning with established criteria for this condition.

### 2.3 Statistical analyses

All statistical analyses were performed using SPSS version 26.0 (IBM Corp., Armonk, NY, United States) and R software version 4.4.0 (R Foundation for Statistical Computing, Vienna, Austria). The nomogram was constructed using the rms package in R. First, a dataset containing the selected independent risk factors identified through multivariate logistic regression was established. The logistic regression model was then fitted using the lrm() function in the rms package. Based on the regression coefficients, the nomogram was generated using the nomogram() function, which visually represents the contribution of each variable to the predicted probability.

The normality of continuous variables was assessed using the Kolmogorov–Smirnov test. Based on the data distribution, continuous variables were either expressed as mean ± standard deviation or median with interquartile range. Categorical variables were presented as percentages (%). Comparative analyses between two groups were conducted using the t-test, Chi-square test, or Mann–Whitney U test, as appropriate. Significant variables from the univariate analysis were selected for inclusion in a multivariate binary logistic regression model, which was used to construct the nomogram. The nomogram uses a Linear Predictor component to map the Total Points to a probability. The Total Points, calculated by summing the points for each variable, is then input into a linear regression model that provides the predicted probability of the outcome. This mapping allows clinicians to easily interpret the total score as a probability, facilitating informed decision-making. Receiver operating characteristic (ROC) curves were generated to assess the discriminatory ability of the nomogram. Calibration curves were plotted to compare the observed and predicted probabilities. Decision curve analysis (DCA) was employed to evaluate the clinical utility of the model by estimating its net benefit for clinical decision-making. A *p* value of less than 0.05 was considered statistically significant.

## 3 Results

### 3.1 Clinical findings

Between January 1, 2014 and May 31, 2023, a total of 292 patients were enrolled. Pathological diagnoses revealed 164 cases of DN and 128 cases of NDN. The clinical manifestations of these groups are detailed in Table 1. No significant differences were observed between DN and NDN patients in terms of gender. Patients with NDN were older and had a higher body mass index (BMI). Patients with DN were found to have a higher prevalence of comorbid conditions, including hypertension, renal insufficiency, coronary heart disease, and diabetic retinopathy, when compared to those with NDN. Furthermore, DN patients exhibited notably higher median levels of hemoglobin A1c (HbA1c) (7.75 mmol/L *vs.* 6.40 mmol/L, *p* < 0.001), fasting blood glucose (6.72 mmol/L vs. 5.86 mmol/L, *p* < 0.001), proteinuria (4.21 g/24 h vs. 3.00 g/24 h, *p* = 0.001), and neutrophil-to-lymphocyte ratio (NLR) (3.30 vs. 2.62, *p* < 0.001). Additionally, these patients had significantly lower eGFR (median, 53.16 mL/min/1.73 m^2^ vs. 79.77 mL/min/1.73 m^2^, *p* < 0.001), and their kidney sizes were significantly larger (median, 180.18 cm^3^ vs. 149.02 cm^3^, *p* < 0.001).

**Table 1:** Clinical findings.

Characteristic	DN (*n* = 164)	NDN (*n* = 128)	*p*
Male sex, (*n*)	116 (70.7%)	82 (64.1%)	0.226
Age (y)	54 (46~60)	62 (52~68)	<0.001
BMI (kg/m^2^)	23.65 (21.91~25.68)	24.30 (22.73~26.38)	0.006
Hypertension (*n*)	144 (87.8%)	83 (64.8%)	<0.001
Diabetic retinopathy (*n*)	140 (85.4%)	22 (17.2%)	<0.001
Coronary heart disease (*n*)	16 (9.8%)	2 (1.6%)	0.004
Pleural effusion (*n*)	62 (37.8%)	25 (19.5%)	0.001
Pericardial effusion (*n*)	43 (26.2%)	12 (9.4%)	<0.001
Duration of diabetes (m)	108 (60~144)	24 (6~81)	<0.001
Duration of kidney disease (m)	9 (4~24)	5 (1~12)	<0.001
SBP (mmHg)	148 (135~162)	134 (120~150)	<0.001
DBP (mmHg)	83 (75~95)	79 (73~89)	0.023
HbA1c (%)	7.75 (6.53~9.10)	6.40 (6.00~7.35)	<0.001
Fasting blood glucose (mmol/L)	6.72 (4.84~8.98)	5.86 (4.65~7.03)	0.006
Scr (μmol/L)	129.00 (96.15~175.53)	84.90 (61.00~126.95)	<0.001
eGFR (mL/min/1.73 m^2^)	53.16 (37.11~74.52)	79.77 (46.17~97.78)	<0.001
Urea nitrogen (mmol/L)	9.11 (6.58~13.99)	6.89 (4.93~9.60)	<0.001
Uric acid (μmol/L)	382.00 (326.80~434.30)	383.20 (319.05~456.63)	0.605
Serum albumin (g/L)	29.00 (25.20~34.30)	28.90 (22.50~37.40)	0.730
Proteinuria (g/24 h)	4.21 (2.50~7.41)	3.00 (1.15~6.12)	0.001
Hematuria (*n*)	133 (81.1%)	109 (85.2%)	0.361
Urinary RBCs (/HPF)	4.00 (1.94~9.59)	8.57 (2.09~27.12)	0.006
Hemoglobin (g/L)	107.1 ± 24.3	124.2 ± 25.3	0.289
WBC (× 10^9^/L)	6.80 (5.67~8.14)	6.85 (5.84~8.80)	0.272
NLR	3.30 (2.42~4.45)	2.62 (1.80~3.67)	<0.001
Platelet (× 10^9^/L)	241.0 0 (196.50~305.00)	254.50 (193.50~302.75)	0.943
Triglycerides (mmol/L)	1.63 (1.12~2.45)	2.06 (1.30~2.90)	0.009
Total cholesterol (mmol/L)	5.34 (4.29~6.86)	5.65 (4.26~8.07)	0.182
Serum phosphate (mmol/L)	1.27 (1.08~1.43)	1.21 (1.05~1.32)	0.012
Immunoglobulin G (g/L)	9.26 (6.91~10.90)	8.80 (5.69~11.58)	0.405
Immunoglobulin A (g/L)	2.63 (1.93~3.26)	2.52 (1.98~3.55)	0.885
Immunoglobulin M (g/L)	0.95 (0.66~1.17)	1.02 (0.71~1.43)	0.038
Length of the left kidney (cm)	12.60 (11.00~12.60)	11.02 (10.65~11.02)	<0.001
Length of the right kidney (cm)	11.20 (11.00~11.20)	10.72 (10.39~10.72)	<0.001
Kidney volume (cm^3^)	180.18 (150.23~180.18)	149.02 (138.13~149.02)	<0.001
RAAS inhibitors (*n*)	69 (42.1%)	49 (38.3%)	0.512
SGLT2 inhibitors (*n*)	23 (14.0%)	13 (10.2%)	0.318

DN, diabetic nephropathy; NDN, non-diabetic nephropathy; BMI, body mass index; SBP, Systolic blood pressure; DBP, diastolic blood pressure; HbA1c, hemoglobin A1c; Scr, serum creatinine; RBC, red blood cell count; WBC, white blood cell count; NLR, neutrophil-to-lymphocyte ratio.

### 3.2 Pathological findings

The renal histopathological classifications are detailed in Table 2. In our study, NDN included several types, with MN being the most common, comprising 52 (40.6%) cases. This was followed by IgA nephropathy and minimal change disease, with 17 (13.3%) and 10 (7.8%) cases, respectively. It is noteworthy that during the process of re-examining the slides, we identified cases of NDN that had been misdiagnosed as DN. For instance, one case of light chain deposition disease was discovered.

**Table 2:** Pathological types of NDN.

Pathological types	Case (%)
Membranous nephropathy	52 (40.6%)
IgA nephropathy	17 (13.3%)
Minimal change disease	10 (7.8%)
Minor glomerular abnormalities	7 (5.5%)
ANCA associated glomerulonephritis	7 (5.5%)
HBV-associated glomerulonephritis	6 (4.7%)
Lupus nephritis	6 (4.7%)
Hypertensive nephrosclerosis	5 (3.9%)
Mesangial proliferative glomerulonephritis	4 (3.1%)
Membranoproliferative glomerulonephritis	3 (2.3%)
Focal segmental glomerulonephritis	2 (1.6%)
Henoch-Schonlein purpura nephritis	2 (1.6%)
Obesity-related glomerulopathy	1 (0.8%)
Type II crescentic glomerulonephritis	1 (0.8%)
Malignancy-associated membranous nephropathy	1 (0.8%)
Light chain cast nephropathy	1 (0.8%)
Amyloid immunoglobulin light chain amyloidosis	1 (0.8%)
Light chain deposition disease	1 (0.8%)
IgG4-related renal disease	1 (0.8%)

### 3.3 Variable screening

Univariate analysis revealed several factors associated with the diagnosis of NDN, including the duration of diabetes, HbA1c, eGFR, and kidney volume. Concurrently, the presence of diabetic retinopathy, hypertension, coronary heart disease, and serous cavity effusion are linked with DN (Supplementary Table S1). According to results presented in Table 3, stepwise backward multivariate regression analysis identified independent risk factors for NDN. These include the presence of diabetic retinopathy, duration of diabetes, HbA1c, systolic blood pressure (SBP), NLR, kidney volume, triglycerides, eGFR, and urinary red blood cell count (urinary RBCs).

**Table 3:** Multivariate logistic regression analysis of risk factors for DN.

Risk factors	*β* value	S. E	*p*	OR	95 CI
Diabetic retinopathy	2.419	0.411	<0.001	11.235	5.019~25.145
Duration of diabetes	0.011	0.004	0.003	1.011	1.004~1.018
HbA1c	0.523	0.138	<0.001	1.688	1.288~2.211
Systolic blood pressure	0.022	0.009	0.011	1.022	1.005~1.040
NLR	0.072	0.033	0.029	1.074	1.007~1.146
Kidney volume	0.041	0.009	<0.001	1.042	1.024~1.059
Triglycerides	−0.264	0.119	0.027	0.768	0.608~0.970
eGFR	−0.013	0.006	0.034	0.987	0.975~0.999
Urinary RBCs	−0.001	0.001	0.048	0.999	0.997~0.999

NLR, neutrophil-to-lymphocyte ratio, S. E, Standard error, OR, Odds ratio, CI, Confidence interval.

### 3.4 Model building

**Figure 2:**
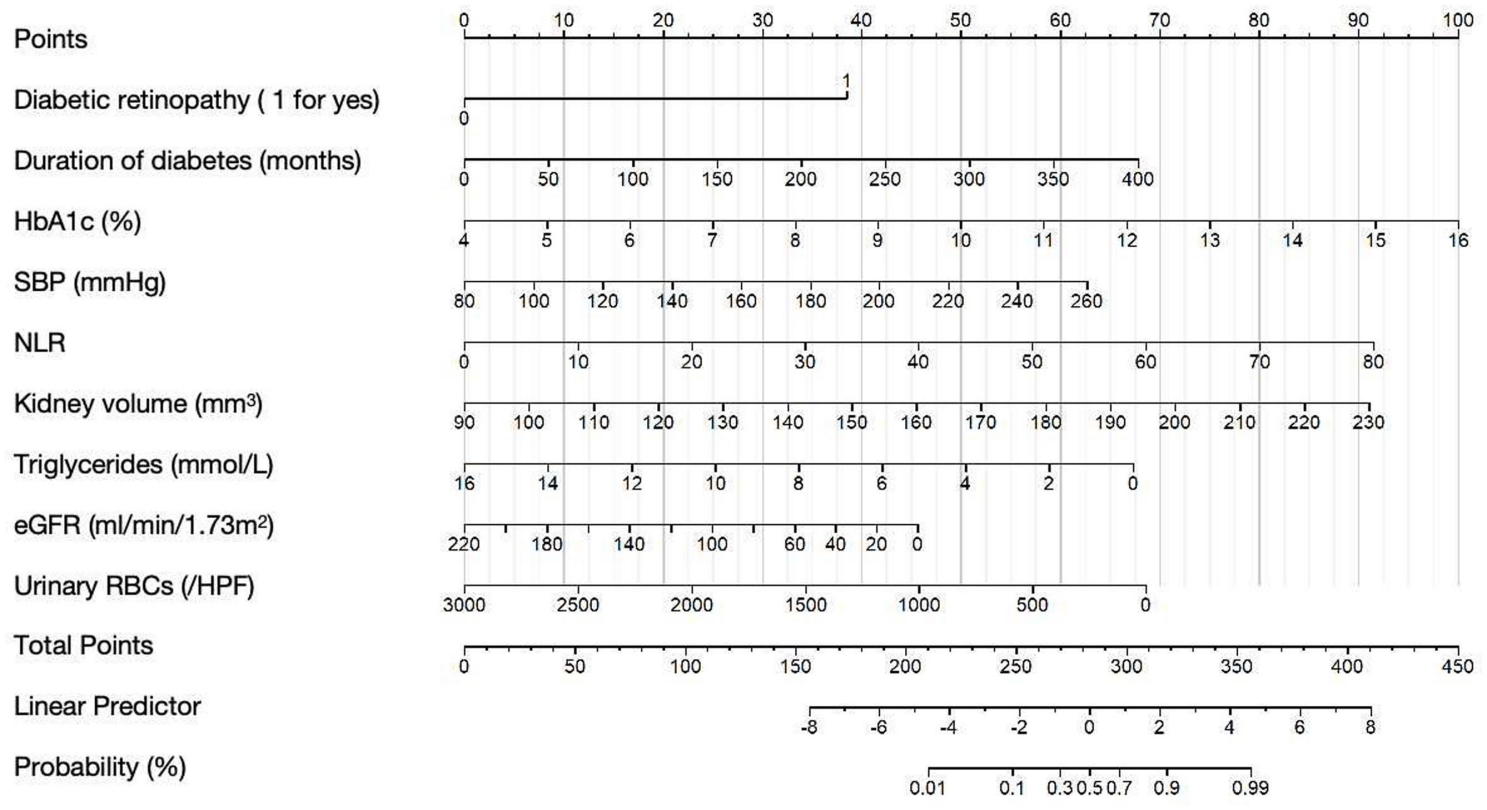
Nomogram for predicting DN in T2DM patients. Example Calculation: For a patient with the following characteristics: Diabetic retinopathy: yes (score = 38 points), Duration of diabetes: 50 months (score = 8 points), HbA1c: 10.0% (score = 50 points), SBP: 140 mmHg (score = 22 points), NLR: 30 (score = 34 points), Kidney volume: 140 mm3 (score = 32 points), Triglycerides: 10 mmoL/L (score = 25 points), eGFR: 60 mL/min/1.73 m2 (score = 33 points), Urinary RBCs: 500/ul (score = 57 points); Total points = 38 + 50 + 25 + 22 + 34 + 32 + 25 + 22 + 57 = 305; The corresponding predicted probability of NDN is approximately 80%.

According to the results from the multivariate regression analysis, we developed a nomogram to predict the risk of DN in T2DM patients (Figure 2). The nomogram incorporates nine predictive variables: the presence of diabetic retinopathy, duration of diabetes (months), HbA1c, SBP, NLR, kidney volume, triglycerides, eGFR, and urinary RBCs, each assigned a specific score based on its weight. To use the nomogram, identify each variable’s value on the corresponding axis, draw a line upward to the points scale, and sum the scores. The total score is then mapped to the diagnostic probability, providing an estimate of the likelihood of DN.

Collinearity among predictors was assessed using correlation analysis and sensitivity testing. The correlation coefficients between variables were all below 0.3, indicating no significant collinearity (Supplementary Figure S1). Sensitivity analysis further confirmed that the exclusion of any individual predictor did not substantially affect the regression coefficients of the remaining variables, suggesting model stability (Supplementary Table S2).

In cases where missing values were present in the variables used for the nomogram, we recommend using median imputation based on the data in our Table 1 to help assist in decision-making. This approach was used to handle the missing data while preserving the overall distribution of the variables.

### 3.5 Model evaluation

**Figure 3:**
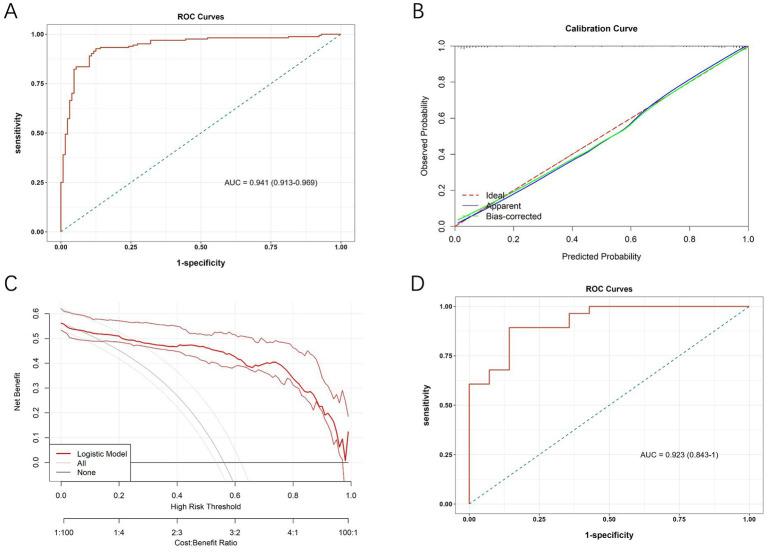
**(A)** ROC curve of the diagnostic model in the initial cohort. **(B)** Calibration curves of the nomogram. **(C)** Decision curve analysis curves of the nomogram. **(D)** ROC curve of the diagnostic model in the validation cohort.

The diagnostic value of the developed differential diagnostic model was assessed using ROC curves, calibration curves, and DCA. The model demonstrated a high diagnostic accuracy with an AUC of 0.941 (95% CI, 0.913~0.969; Figure 3A). The calibration curves at different time points demonstrated a strong alignment between predicted and actual outcomes (Figure 3B). Figure 3C presents the DCA curves, which assess the net clinical benefit of the nomogram’s predictions across different risk thresholds. Net benefit was highest across thresholds ranging from 6% to 94%, indicating that the nomogram provides the greatest clinical utility within this range. The optimal threshold, corresponding to the model’s cut-off value, was approximately 48.9%. The red line consistently remained above both the “ALL” and “None” lines, suggesting that interventions guided by the nomogram offer a favorable clinical advantage and support more informed decision-making.

A sensitivity analysis was conducted to assess the robustness of predictive performance of the model by sequentially removing individual predictors and observing the impact on AUC (Supplementary Figures S2, S3). The full model achieved an AUC of 0.941, while removal of diabetic retinopathy led to the greatest reduction in AUC (by 0.024), highlighting its critical role. Excluding other variables, such as eGFR and triglycerides, resulted in only minor changes in AUC, but these variables were retained considering their clinical relevance.

In addition, we calculated the Youden’s index to determine the optimal threshold for sensitivity and specificity in clinical use. The maximum Youden’s index of 0.802 was achieved when the predicted probability was 0.489, which served as the cut-off for differentiating DN from NDN. Based on this threshold, a predicted probability of ≥0.489 indicates DN, while <0.489 suggests NDN. This threshold was selected to maximize the balance between sensitivity and specificity, providing clinicians with a reliable decision-making tool.

To further assess its diagnostic efficacy after development, we collected data from T2DM patients who underwent renal biopsy from June 1, 2023, to December 31, 2023, at the aforementioned three hospitals for external validation. The external validation cohort included 42 patients, with 28 diagnosed with DN and 14 with NDN. The baseline demographic and clinical characteristics of patients in the validation cohort, stratified by pathological diagnosis, are summarized in Supplementary Table S3. The results, presented in Figure 3D, showed an AUC of 0.923 (95% CI, 0.843~1.000) for the ROC analysis.

**Figure 4:**
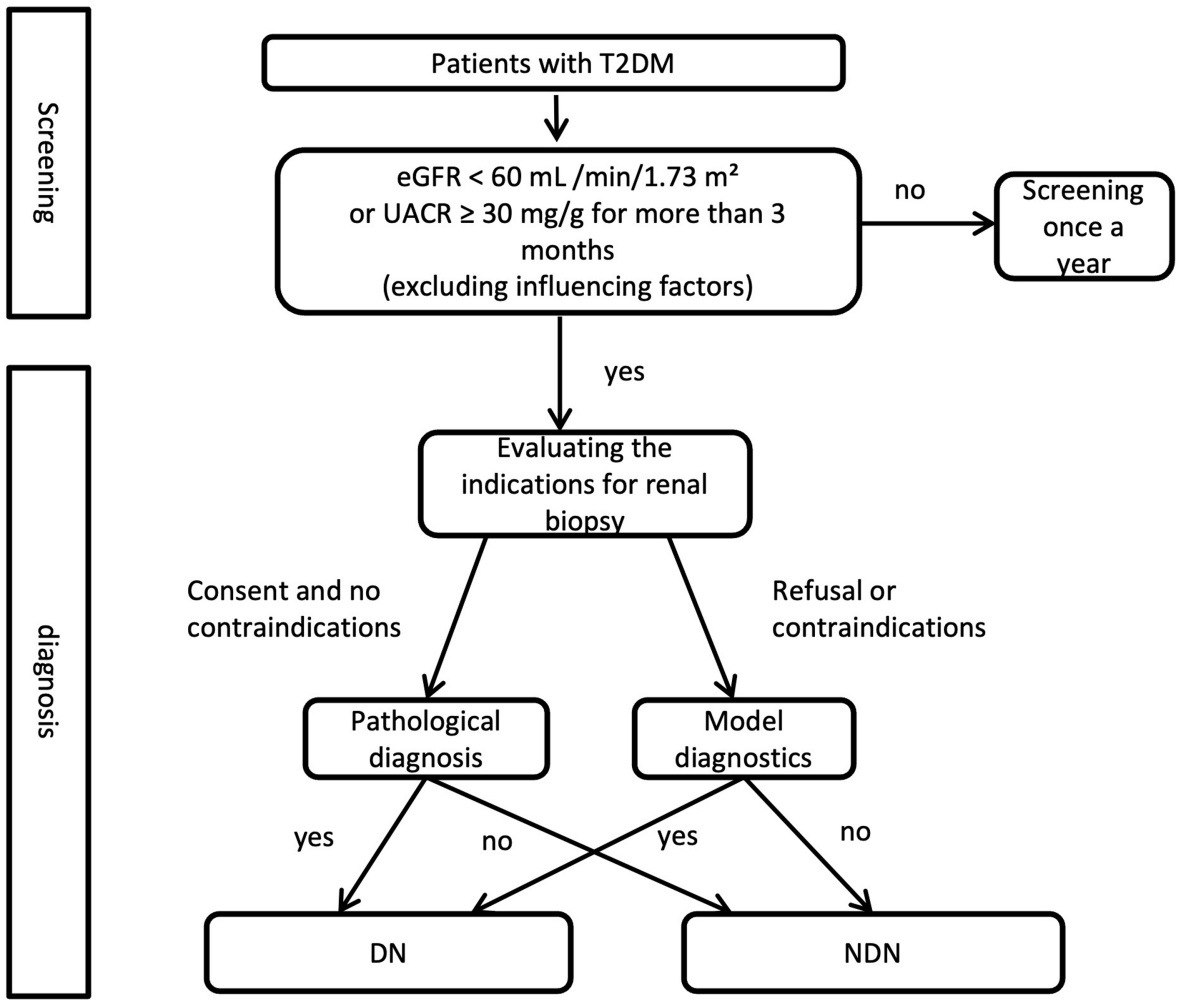
Clinical decision flowchart for renal biopsy vs. nomogram use in T2DM with renal impairment.

A clinical decision flowchart (Figure 4) was developed to assist clinicians in determining when to use the nomogram vs. proceeding directly with renal biopsy. The nomogram is recommended for patients with T2DM and renal impairment (eGFR <60 mL/min/1.73 m^2^ or UACR ≥ 30 mg/g) when biopsy is contraindicated or declined. For patients without contraindications, renal biopsy remains the gold standard for pathological diagnosis. This strategy offers a non-invasive option for risk assessment while maintaining diagnostic accuracy, supporting more precise differentiation between DN and NDN based on clinical and biochemical markers.

## 4 Discussion

DN is acknowledged as one of the most common causes of end-stage renal disease globally. Surprisingly, more than half of diabetic patients with kidney disease do not actually suffer from DN (7). Accurate differentiation between DN and NDN is vital for diabetic patients, as these conditions require significantly different treatment and management strategies (8, 18). Currently, renal biopsy is the gold standard for diagnosing DN, yet not all patients are eligible for this invasive procedure. In patients with T1DM, the duration of diabetes, proteinuria, and diabetic retinopathy are reliable predictive indicators for DN. However, their specificity declines in patients with T2DM (18, 19). Therefore, the development of a non-invasive, convenient, and precise diagnostic method specifically for T2DM patients holds substantial importance for both clinicians and patients.

In our cohort, a total of 292 patients were enrolled, with 128 (43.7%) diagnosed with NDN. This proportion is similar to that observed in previous studies (10–12). Among these NDN patients, MN was the most prevalent renal pathology, comprising 40.6% (52/128) of cases, which is higher than rates reported in earlier research (11, 13). IgA nephropathy was the second most common condition, accounting for 13.3%. This is in contrast to prior studies, which identified IgA nephropathy as the most prevalent NDN among diabetic patients, with an incidence rate of about 35% (11, 20). This discrepancy may be attributed to the increasing incidence of MN, potentially linked to air pollution (21, 22). Overall, MN and IgA nephropathy remain the most common types of NDN among patients with T2DM, aligning with patterns observed in the general population.

Our study reveals that patients with NDN are older and exhibit higher BMI compared to those with DN, contradicting prior studies which found no significant age differences between these groups (12, 23). This discrepancy may be attributed to the high prevalence of MN within our NDN cohort, a condition more common among the elderly. Studies have shown that obesity contributes to kidney outcomes in patients with glomerular diseases, including MN (24, 25). For example, a study by Chen et al. found that in a cohort of 200 MN patients, obesity (BMI ≥ 30 kg/m^2^) was associated with a 40% increase in glomerular injury, measured by declines in GFR and urinary albumin levels (24). Additionally, BMI ≥ 30 kg/m^2^ was associated with increased mesangial lesions and significant mesangial matrix expansion in idiopathic MN (24). This association is thought to be mediated by TGF-β1, a cytokine involved in glomerular injury and fibrosis. These findings emphasize the potential role of obesity in the progression of MN, which could explain the higher BMI observed in our NDN cohort. These findings underscore the importance of considering BMI as a key factor in diagnosing and managing NDN, as it may be indicative of underlying MN, especially in patients with T2DM. The inclusion of BMI in future diagnostic models could provide more accurate predictions of renal outcomes in these patients.

Consistent with earlier findings, patients with DN more frequently suffer from comorbid conditions such as hypertension and coronary heart disease, likely due to the prolonged duration of diabetes associated with DN (11, 13). Additionally, our results show a higher susceptibility to serous cavity effusions among DN patients. In our study, the level of proteinuria in the DN group was significantly higher than in the NDN group, while there were no significant differences in the prevalence of hematuria between the two groups. However, we found that the urinary RBCs was higher in the NDN group compared to the DN group, and in multivariate analysis, urinary RBCs was established as an independent risk factor, consistent with previous findings. This is likely because urinary RBCs, being a continuous variable, provide a more precise measurement of renal injury, whereas hematuria is a categorical variable. Additionally, a retrospective analysis confirmed that, in T2DM patients, dysmorphic red blood cells are a more reliable indicator of NDN than hematuria (26).

The diagnostic model we developed identifies several key factors associated with the onset of DN in patients with T2DM. These include the presence of diabetic retinopathy, the duration of diabetes, HbA1c, SBP, NLR, kidney volume, triglycerides, eGFR, and urinary RBCs. Diabetic retinopathy, diabetes duration, and HbA1c are well-recognized indicators for diagnosing DN, as evidenced by previous studies (10–14). Diabetic retinopathy is one of the most common microvascular complications of diabetes and is closely associated with the severity of systemic microvascular damage. The presence of diabetic retinopathy indicates widespread endothelial dysfunction and microvascular injury, which are also key factors in the pathogenesis of DN (27, 28). Patients with diabetic retinopathy are more likely to have kidney involvement due to shared pathogenic mechanisms, including chronic hyperglycemia and inflammatory processes. The duration of diabetes is a critical factor in the development of DN. Longer diabetes duration increases the cumulative exposure to hyperglycemia, which accelerates the development of nephropathy. Chronic hyperglycemia promotes the formation of advanced glycation end-products, leading to kidney damage through mechanisms such as inflammation, fibrosis, and thickening of the glomerular basement membrane (29). As the duration of diabetes increases, the risk of developing DN also rises. HbA1c is a well-established marker for long-term glycemic control. Elevated HbA1c levels indicate poor control of blood glucose. Studies have consistently shown that maintaining HbA1c levels within target ranges reduces the risk of DN, making it an important independent risk factor for the onset of nephropathy (27).

In our study, SBP was identified as an independent risk factor, as previous report (27). Hypertension is a major risk factor for the development and progression of DN. High systolic blood pressure contributes to glomerular hypertension, which damages the glomerular capillaries, promotes proteinuria, and accelerates the decline in kidney function. Effective management of blood pressure is essential in preventing or slowing the progression of DN, and SBP is a key independent factor in the development of kidney disease in T2DM patients (30, 31). Additionally, NLR, a cost-effective biomarker for subclinical inflammation, has been linked to glucose intolerance and insulin resistance in T2DM and is associated with early organ damage, including DN, which our study also supports (32–34). In T2DM patients, changes in kidney volume, including enlargement or fibrosis, are early indicators of kidney injury. Increased kidney volume often correlates with glomerular hyperfiltration and nephron loss, which are characteristic of the early stages of DN. Kidney enlargement is often seen in patients with impaired kidney function, and it can serve as a useful biomarker for identifying individuals at high risk of developing DN.

Dyslipidemia, particularly elevated triglyceride levels, has been linked to kidney injury in T2DM patients. High triglyceride levels contribute to endothelial dysfunction, increased oxidative stress, and the formation of glomerulosclerosis, which all promote the development of nephropathy. Elevated triglycerides are commonly observed in patients with metabolic syndrome and are strongly associated with the progression of DN (35). The negative association between triglycerides and DN observed in our study may reflect complex metabolic-endocrine interactions. Recent studies have demonstrated that metabolic hormones, such as GLP-1 receptor agonists (GLP-1 RAs), can influence thyroid function and lipid homeostasis in patients with T2DM (36). These interactions may lead to changes in lipid profiles, including triglycerides, which could indirectly impact renal and cardiovascular health. Specifically, GLP-1 RAs can improve lipid metabolism, potentially reducing triglyceride levels, while also influencing thyroid function, a key regulator of metabolic processes (36). In the context of diabetes, changes in lipid profiles might reflect compensatory or treatment-related changes rather than a direct causal link to DN. These findings highlight the complexity of lipid metabolism in T2DM and suggest that triglycerides, while negatively correlated with DN in our study, may be influenced by multiple metabolic factors rather than a straightforward relationship with kidney damage. Moreover, the recently proposed cardiovascular-kidney-metabolic syndrome, characterized by the coexistence of cardiovascular disease, chronic kidney disease, and metabolic disorders, emphasizes the need for a holistic approach to understanding lipid metabolism in T2DM patients (37). The interconnectivity of these conditions highlights the complexity of lipid metabolism and its potential impact on renal and cardiovascular health.

Tubular injury biomarkers, such as neutrophil gelatinase-associated lipocalin (NGAL) and β2-microglobulin, can identify early renal injury, even in the absence of elevated serum creatinine levels (10, 38). Recent studies have shown that hyperbilirubinemia, even as a transient systemic insult, has been proven to cause renal injury, detectable through biomarkers such as NGAL and β2-microglobulin, despite normal serum creatinine levels (38). This finding highlights the potential of next-generation biomarkers in detecting early renal injury and improving the predictive accuracy of diagnostic models. Previous study reported that tubular biomarkers can enhance the level of non-invasive diagnosis of DN (10). However, in our study, most patients had already been diagnosed with diabetes and had experienced some degree of kidney damage by the time they sought care, so NGAL and β2-microglobulin were not widely tested in our cohort. Nonetheless, we believe that future studies should include patients at earlier stages and incorporate these biomarkers to further refine our nomogram, providing better insights into early renal injury and improving the model’s generalizability.

Previous studies have also focused on the differential diagnosis between DN and NDN in patients with diabetes. However, most of these were single-center studies with smaller sample sizes, fewer included variables, and often utilized non-visualized models (10–15). For example, Wang et al. (10) included 132 diabetic patients who underwent renal biopsy at a single center and focused on tubulointerstitial markers, finding that NGAL and β2-microglobulin could improve the diagnostic accuracy for DN. Similarly, Zhao et al. (12) developed a risk score model for differential diagnosis, identifying diabetic retinopathy, diabetes duration ≥ 5 years, eGFR < 30 mL/min/1.73 m^2^, 24-h urinary protein ≥ 3 g, and absence of hematuria as independent risk factors for DN. However, their model was non-visualized and showed relatively lower performance, with an AUC of only 0.86. Jiang et al. also developed a nomogram that included variables such as gender, diabetes duration, diabetic retinopathy, hematuria, HbA1c, anemia, blood pressure, urinary protein excretion, and eGFR. However, in their study, all variables were treated as categorical, which reduced the model’s precision. For example, HbA1c was dichotomized as greater than or less than 7%, and blood pressure was categorized based on hypertension classification, which likely imited its precision. Moreover, they did not provide ROC curve analysis to assess the model’s diagnostic performance (14). Zhou et al. constructed a machine learning model to predict DN but included only 100 T2DM patients who underwent renal biopsy. Their logistic regression model incorporated variables such as the triglyceride-to-cystatin C ratio, systolic blood pressure, diabetes duration, diabetic retinopathy, HbA1c, and hemoglobin. The relatively small sample size and limited external validation restrict the generalizability of their model (15). Compared with these studies, our model offers several advantages. It is based on a multicenter cohort with a larger sample size, incorporates nine routinely available clinical variables that enhance its practicality, and is presented as a visualized nomogram, which improves interpretability and clinical usability. Moreover, it demonstrates higher diagnostic performance, with an AUC of 0.941 in the derivation cohort and 0.923 in the validation cohort, further supporting its reliability.

Our nomogram, which uses easily accessible clinical and laboratory variables, has significant potential for integration into routine clinical practice. It serves as an adjunct tool to help clinicians make informed decisions about the need for a renal biopsy, particularly when NDN is suspected. This model is especially valuable in settings where renal biopsy may not be feasible due to resource constraints, enabling clinicians to make more rational diagnostic decisions. Its integration into clinical workflows could aid in the early detection and management of NDN, ultimately improving patient outcomes. While the model is helpful for guiding biopsy decisions, it is not representative of the entire T2DM population and does not replace renal biopsy, which remains the gold standard for diagnosing NDN.

We acknowledge certain limitations of this study, including the incomplete accounting for treatment factors, selection bias, the small sample size, especially in the validation cohort, and the geographical restriction to Fujian Province, which may not represent the broader population. This may limit the generalizability of the model to other ethnic groups or healthcare systems. Factors such as lifestyle, genetic differences, and treatment-related factors could influence the applicability of the model in different regions or populations. Moreover, the model’s performance may vary in populations with different prevalences of NDN, particularly in regions where MN is less common, such as in Western cohorts. This geographical and demographic variation in disease distribution may impact the generalizability of our model. In this study, the validation cohort was drawn from the same hospitals as the development cohort. Future studies should aim to validate the model using cohorts from different hospitals or regions to more robustly assess its generalizability. The lack of patient follow-up makes it difficult to ascertain whether those diagnosed with NDN developed DN later, or vice versa. Future studies should aim to validate the model in larger, multi-centered cohorts from diverse geographical locations, including follow-ups, to more robustly validate our model.

In conclusion, our study has developed a non-invasive diagnostic model that effectively distinguishes NDN from DN in patients with T2DM. This model could assist physicians in making more informed decisions about renal lesions in T2DM patients when a renal biopsy is not feasible, thereby facilitating more targeted and rational diagnostic approaches.

## Data Availability

The raw data supporting the conclusions of this article will be made available by the authors, without undue reservation.
