# Supplementary material for: Development and validation of a predictive nomogram for differentiating diabetic nephropathy from non-diabetic nephropathy in patients with T2DM: a multicenter study
Source: Front Nutr. 2025 Jun 2;12:1605841. doi: 10.3389/fnut.2025.1605841 (PMC12171117; doi:10.3389/fnut.2025.1605841)
Supplement: Supplementary file 1 [file Data_Sheet_1.docx]

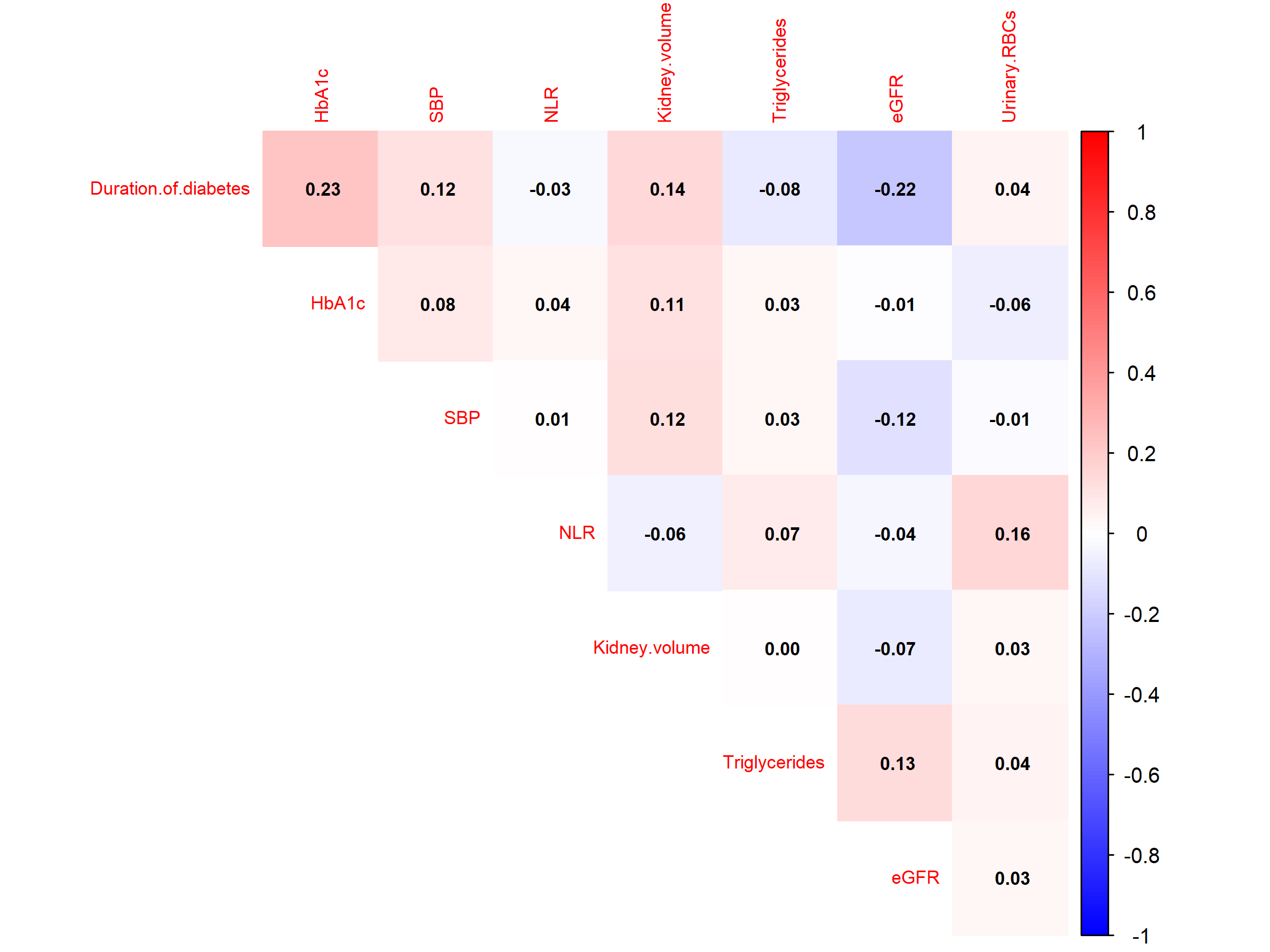


Figure S1. The correlation coefficients (|r|) between all variables.


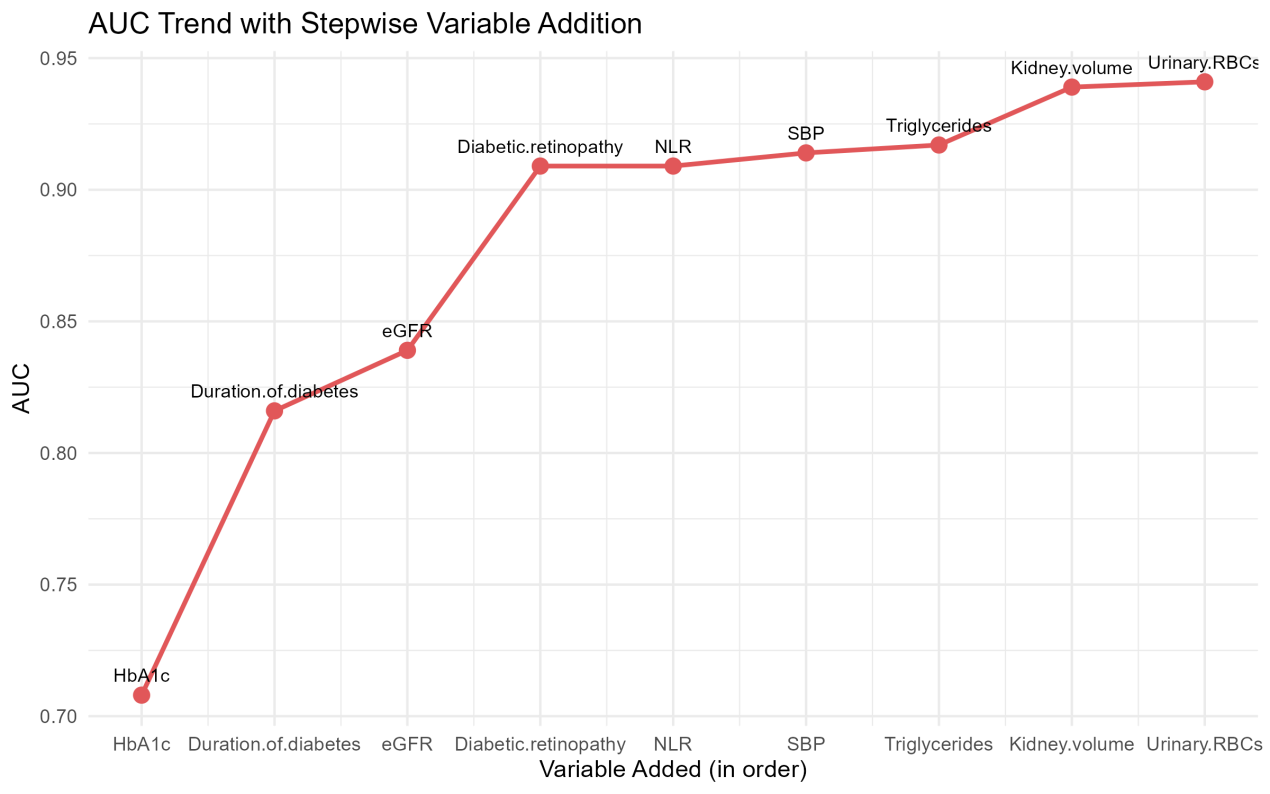


Figure S2: Impact of Sequential Variable Addition on Model Performance


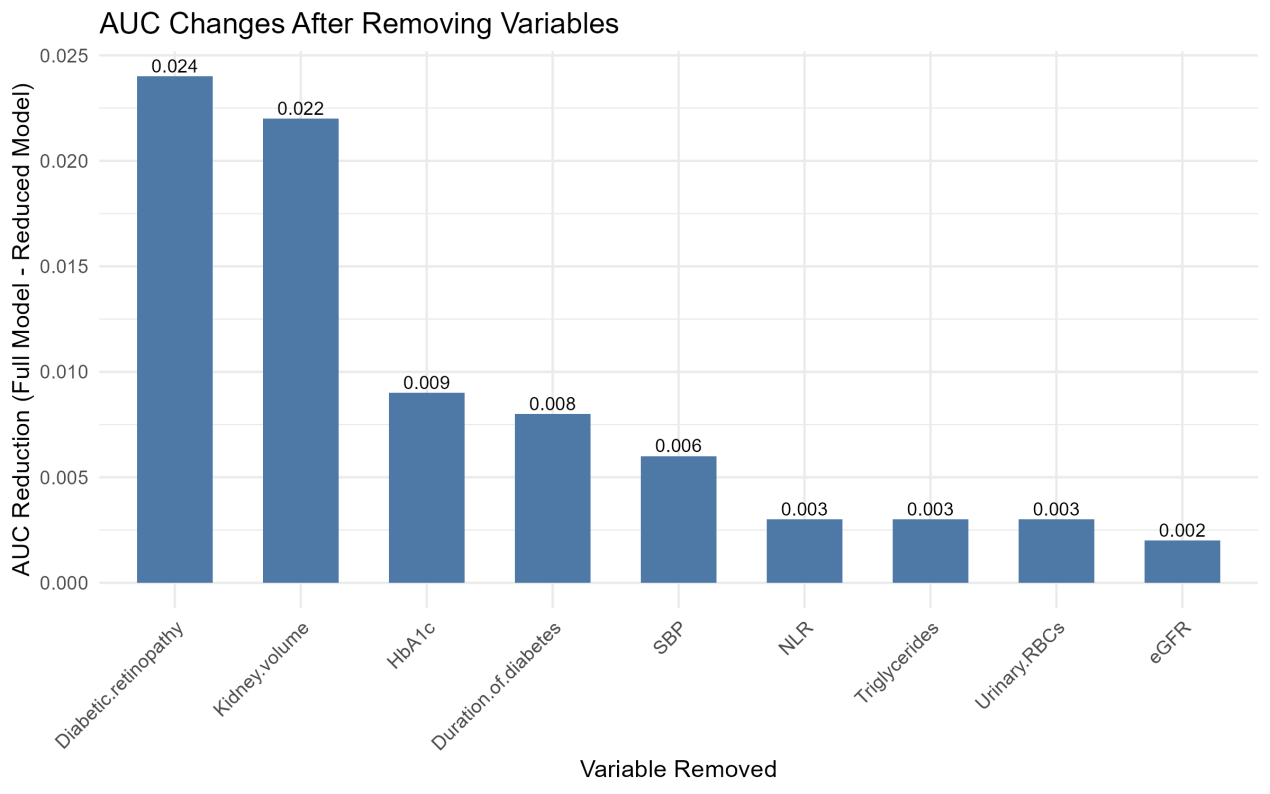


Figure S3: Impact of Sequential Variable Removal on Model Performance

Table S1. Univariate analysis logistic regression analysis of risk factors for DN

| Risk factors | OR | 95% CI | P |
| --- | --- | --- | --- |
| Age (y) | 0.956 | 0.936~0.976 | <0.001 |
| BMI (kg/m^2^) | 0.885 | 0.817~0.958 | 0.003 |
| Hypertension (*n*) | 3.904 | 2.160~7.056 | <0.001 |
| Coronary heart disease (*n*) | 6.811 | 1.536~30.191 | 0.012 |
| Diabetic retinopathy (*n*) | 28.106 | 14.952~52.833 | <0.001 |
| Pleural effusion (*n*) | 2.504 | 1.461~4.293 | 0.001 |
| Pericardial effusion (*n*) | 3.435 | 1.725~6.84 | <0.001 |
| Duration of diabetes (m) | 1.019 | 1.014~1.024 | <0.001 |
| Duration of kidney disease (m) | 1.004 | 0.995~1.013 | 0.410 |
| SBP (mmHg) | 1.025 | 1.014~1.037 | <0.001 |
| DBP (mmHg) | 1.018 | 1.000~1.036 | 0.051 |
| HbA1c (%) | 1.571 | 1.332~1.854 | <0.001 |
| SCr (μmol/L) | 1.004 | 1.001~1.006 | 0.005 |
| BUN (mmol/L) | 1.07 | 1.023~1.118 | 0.003 |
| eGFR (mL/min/1.73 m²) | 0.982 | 0.974~0.989 | <0.001 |
| TG (mmol/L) | 0.860 | 0.752~0.983 | 0.027 |
| UTP (g/24h) | 1.076 | 1.009~1.146 | 0.025 |
| Kidney volume (cm^3^) | 1.041 | 1.029~1.054 | <0.001 |
| NLR | 1.026 | 0.965~1.092 | 0.405 |
| Fasting blood glucose(mmol/L) | 1.168 | 1.073~1.272 | <0.001 |
| Serum phosphate (mmol/L) | 2.824 | 1.109~7.193 | 0.029 |
| Immunoglobulin M (g/L) | 0.626 | 0.423~0.928 | 0.020 |
| Urinary RBCs (/HPF) | 0.999 | 0.998~1.001 | 0.273 |

Table S2. Sensitivity analysis of predictor variables and their impact on regression coefficients

| Removed_Variable | Coef_Changes |
| --- | --- |
| Diabetic retinopathy | Duration.of.diabetes : 69.1 %; HbA1c : -3.7 %; SBP : 20 %; NLR : -14.7 %; Kidney.volume : 20.4 %; Triglycerides : -2 %; eGFR : 27.3 %; Urinary.RBCs : 0 % |
| Duration of diabetes | NA : NA %; HbA1c : 5.5 %; SBP : -13.3 %; NLR : -9.6 %; Kidney.volume : -6.7 %; Triglycerides : -9.3 %; eGFR : 10.9 %; Urinary.RBCs : -19.4 % |
| HbA1c | NA : NA %; Duration.of.diabetes : 10.3 %; SBP : -14.7 %; NLR : -2.3 %; Kidney.volume : -4.2 %; Triglycerides : -22.9 %; eGFR : -22.7 %; Urinary.RBCs : 10.6 % |
| SBP | NA : NA %; Duration.of.diabetes : -7.8 %; HbA1c : -5.5 %; NLR : -3.9 %; Kidney.volume : 0 %; Triglycerides : -13.9 %; eGFR : 2.8 %; Urinary.RBCs : -4.1 % |
| NLR | NA : NA %; Duration.of.diabetes : -6.3 %; HbA1c : -2.8 %; SBP : -4.9 %; Kidney.volume : -6.2 %; Triglycerides : -4.2 %; eGFR : 7.5 %; Urinary.RBCs : -17.9 % |
| Kidney.volume | NA : NA %; Duration.of.diabetes : -13.5 %; HbA1c : -13.5 %; SBP : -5.7 %; NLR : -23 %; Triglycerides : -8.6 %; eGFR : -8.6 %; Urinary.RBCs : -19.3 % |
| Triglycerides | NA : NA %; Duration.of.diabetes : -2.1 %; HbA1c : -7.6 %; SBP : -15.7 %; NLR : -11 %; Kidney.volume : -1.2 %; eGFR : 3.7 %; Urinary.RBCs : 4.3 % |
| eGFR | NA : NA %; Duration.of.diabetes : 3.8 %; HbA1c : -8.8 %; SBP : -0.1 %; NLR : 5.9 %; Kidney.volume : 0 %; Triglycerides : -1.1 %; Urinary.RBCs : 3 % |
| Urinary RBCs | NA : NA %; Duration.of.diabetes : -5.9 %; HbA1c : 1.3 %; SBP : 1.1 %; NLR : -17.6 %; Kidney.volume : -3.8 %; Triglycerides : -1.1 %; eGFR : 7.9 % |

Table S3. Baseline characteristics of the validation cohort

| Characteristic | DN(*n*=28) | NDN(*n*=14) | *P* |
| --- | --- | --- | --- |
| Male sex, (*n*) | 22（78.6%） | 10（71.4%） | 0.608 |
| Age (y) | 55.82±11.69 | 66.93±9.00 | 0.626 |
| Duration of diabetes (m) | 120(120~192) | 60 (2.75~126) | 0.011 |
| Diabetic retinopathy (*n*) | 25(89.3%) | 3(21.4%) | <0.001 |
| HbA1c (%) | 8.00(7.35~8.73) | 6.80(6.50~7.80) | 0.067 |
| SBP (mmHg) | 145.75±23.35 | 142.64±21.00 | 0.513 |
| NLR | 2.72(1.95~3.64) | 2.42(1.93~3.48) | 0.689 |
| Kidney volume (cm^3^) | 137.80(130.00~160.02) | 108.03(106.20~137.15) | 0.007 |
| Triglycerides (mmol/L) | 1.65(1.08~2.32) | 1.88(1.08~2.52) | 0.631 |
| eGFR (mL/min/1.73 m²) | 53.44±27.62 | 72.28±18.99 | 0.216 |
| Urinary RBCs (/HPF) | 1.85(0.36~4.53) | 3.67(0.22~20.32) | 0.298 |
